# Identification of arthropathy and myopathy of the temporomandibular syndrome by biomechanical facial features

**DOI:** 10.1186/s12938-020-00764-5

**Published:** 2020-04-15

**Authors:** Bruno Coelho Calil, Danilo Vieira da Cunha, Marcus Fraga Vieira, Adriano de Oliveira Andrade, Daniel Antônio Furtado, Douglas Peres Bellomo Junior, Adriano Alves Pereira

**Affiliations:** 1 Laboratory of Biomedical Engineering, Faculty of Electrical Engineering, Federal University of Uberlandia, Av. Joao Naves de Avila, 2121, Uberlandia, 38408-100 Brazil; 2 Bioengineering and Biomechanics Laboratory, Federal University of Goias, Av. Esperanca, s/n, Campus Samambaia, Goiania, GO 74690-900 Brazil

**Keywords:** Temporomandibular disorder, TMD, TMJ, KNN, Quantitative assessment

## Abstract

**Background:**

Temporomandibular disorders (TMDs) are pathological conditions affecting the temporomandibular joint and/or masticatory muscles. The current diagnosis of TMDs is complex and multi-factorial, including questionnaires, medical testing and the use of diagnostic methods, such as computed tomography and magnetic resonance imaging. The evaluation, like the mandibular range of motion, needs the experience of the professional in the field and as such, there is a probability of human error when diagnosing TMD. The aim of this study is therefore to develop a method with infrared cameras, using the maximum range of motion of the jaw and four types of classifiers to help professionals to classify the pathologies of the temporomandibular joint (TMJ) and related muscles in a quantitative way, thus helping to diagnose and follow up on TMD.

**Methods:**

Forty individuals were evaluated and diagnosed using the diagnostic criteria for temporomandibular disorders (DC/TMD) scale, and divided into three groups: 20 healthy individuals (control group CG), 10 individuals with myopathies (MG), 10 individuals with arthropathies (AG). A quantitative assessment was carried out by motion capture. The TMJ movement was captured with camera tracking markers mounted on the face and jaw of each individual. Data was exported and analyzed using a custom-made software. The data was used to identify and place each participant into one of three classes using the K-nearest neighbor (KNN), Random Forest, Naïve Bayes and Support Vector Machine algorithms.

**Results:**

Significant precision and accuracy (over 90%) was reached by KNN when classifying the three groups. The other methods tested presented lower values of sensitivity and specificity.

**Conclusion:**

The quantitative TMD classification method proposed herein has significant precision and accuracy over the DC/TMD standards. However, this should not be used as a standalone tool but as an auxiliary method for diagnostic TMDs.

## Background

Pain in the orofacial region is the major cause of temporomandibular disorder (TMD). A person with TMD has certain limitations, since the temporomandibular joint (TMJ) and its association with the masticatory muscles are responsible for performing complex tasks, such as speech and chewing. The main symptoms of TMD include joint cracking sounds, muscle fatigue, and changes in mandibular pattern movements. Increased muscle strain and internal disturbances of the TMJs may affect the range of feasible mandibular movements [1].

The etiology of TMD is still under discussion, however, it is known to be complex and multi-factorial, involving anatomical, occlusal, muscular and psychological factors [2–4]. The hereditary etiological causes include the idiopathic, systemic psychosomatic and psychosocial aspects of TMD. Exogenous etiological factors include trauma and occlusal disorders [5]. Masticatory muscle conditions are defined as myofascial pain with or without minimal opening by the Diagnostic Criteria for TMD (DC/TMD). TMJ disorders are subdivided by DC/TMD into reduced disk joint displacement, disk displacement with limited opening and without limited opening, arthralgia, osteoarthritis and TMJ osteoarthritis [6].

The prevalence of TMD related to pure arthropathy is relatively low (1.61%), when compared to a pure muscular etiology (11.32%) [7]. The low prevalence of arthropathy makes diagnosis even more difficult. The correct diagnosis of the cause of TMD is relevant to the appropriate treatment. Thus, the use of objective tools to measure the movements of the mouth and to discriminate individuals with muscular TMD from those with arthropathic TMD can help in making the correct diagnosis.

The diagnosis of TMD is mainly descriptive and analytical, including the use of questionnaires, surgical and medical measures such as radiography, computed tomography (CT) and magnetic resonance imaging (MRI) [8]. The DC/TMD protocol is the most widely used for diagnosing TMD, as it is considered valid for the detection of any TMD related to pain [6], but this protocol has the need of an experienced professional in order to determine the underlying cause of TMD [8].

To avoid the need for experienced professionals in the diagnosis of TMD, the use of instruments and methods for quantitatively measuring mandibular movement has seen an increase concerning medical procedures, with the goal of providing an additional base for the assessment of musculoskeletal disorders of the stomatognathic system, as well as for monitoring the success of active treatment approaches [9]. A majority of currently available methods for classifying people with TMDs are based on electromyography [10], imaging processing techniques [11, 12] and temporomandibular sounds [13].

In this sense, Santana-Mora et al. [14] compared surface electromyography (EMG) recorded from the right and left masseter, and temporalis muscles of chronic TMD patients and healthy individuals during resting and clenching. The maximum accuracy, sensitivity and specificity were, respectively, 67%, 69.8% and 84.2%. Haghnegahdar et al. [12] proposed the detection of TMD using image-processing techniques, based on the fact that TMD can manifest itself through changes in bone structure.

Another method used is the active range of motion (AROM) test. This test measures the maximum opening of the mouth, the maximum left and right laterality and the maximum protrusion using a Boley meter or the TheraBite range of motion scale. In this method, the mouth opening is usually measured as the distance between the incisors [15].

Although quantitative methods have some advantages over qualitative methods, these methods have some limitations. The method using surface EMG, presented by Santana-Mora et al. [14], has a low sensitivity and specificity. Diagnostic imaging techniques (radiography, CT and MRI) are often limited by the anatomy of the region and by distortions [16]. Regarding the AROM method, a dentist should interpret all measurements in order to avoid error when interpreting the data. In addition, deviations in mandibular functional movements may be a sign of pathology in the orofacial region. Variables such as the maximum opening capacity of the mouth, however, do not allow for the discrimination of the underlying cause of TMD [17]. Although these methods are used in research that aims to distinguish patients with TMD from healthy individuals, there is a lack of research about differentiating muscular from arthropathic TMD.

To fill the existing gap in research to identify TMD (muscular or arthropathic), the analysis of biomechanical features is a good candidate, as mandibular biomechanical behavior changes with TMD [18]. In addition, animal studies have indicated that TMJ can adapt to changes in biomechanical stress, allowing the joint to maintain an efficient function in the presence of TMD [19]. The detection of these biomechanical changes in mandibular movement can lead to an understanding as to which effects these factors have on TMD. Thus, TMD and its variations (myopathy and arthropathy) can be manifested and detected through changes and adaptations of biomechanical features in mandibular movement.

For the analysis of biomechanical features, in addition to those used by AROM test, it is necessary to track the mandibular movement. Optoelectronic systems for the recording of jaw movements are most frequently used as these are less invasive, while providing accurate and reliable records of mandibular motion, within the linear error margin, which ranges from 0.1 to 1.0 mm [20–22]. The advantage of optoelectronic systems resides in the fact that the markers can be placed at specific points on the head and mandible, allowing for a three-dimensional reconstruction of jaw movement from the markers, including linear trajectory, velocity and acceleration, along with maximum distance.

From movement tracking, it is possible to extract the biomechanical features that will be used for the classification of TMD. The use of predictors such as Random Forest (RF) [23], support vector machine (SVM) [24], Naïve Bayes (NB) [25] and the k-nearest neighbor [26], has helped to classify TMDs. Research by Haghnegahdar et al. [12] used these classifiers for the classification of patients with TMD from healthy individuals. The authors concluded that the KNN method presented the best results in terms of accuracy (92.42%), sensibility (94.70%) and specificity (90.15%).

In this scenario, given the difficulty of diagnosing TMD, and the wide variety of approaches used to test it, the introduction of non-invasive identification methods to support the diagnosis of TMD is an area of valid investigation [27]. This study proposes the analysis of biomechanical features achieved through the trajectory of mandibular movements, collected by an optoelectronic system to record jaw movements as a diagnostic tool for the evaluation of TMD. These features discriminate biomechanical features extracted from individuals with muscular TMD, from those with arthropathic TMD, using the classifiers K-Nearest Neighbor—KNN, Support Vector Machine, Naïve Bayes and Random Forest [12].

## Results

**Fig. 1:**
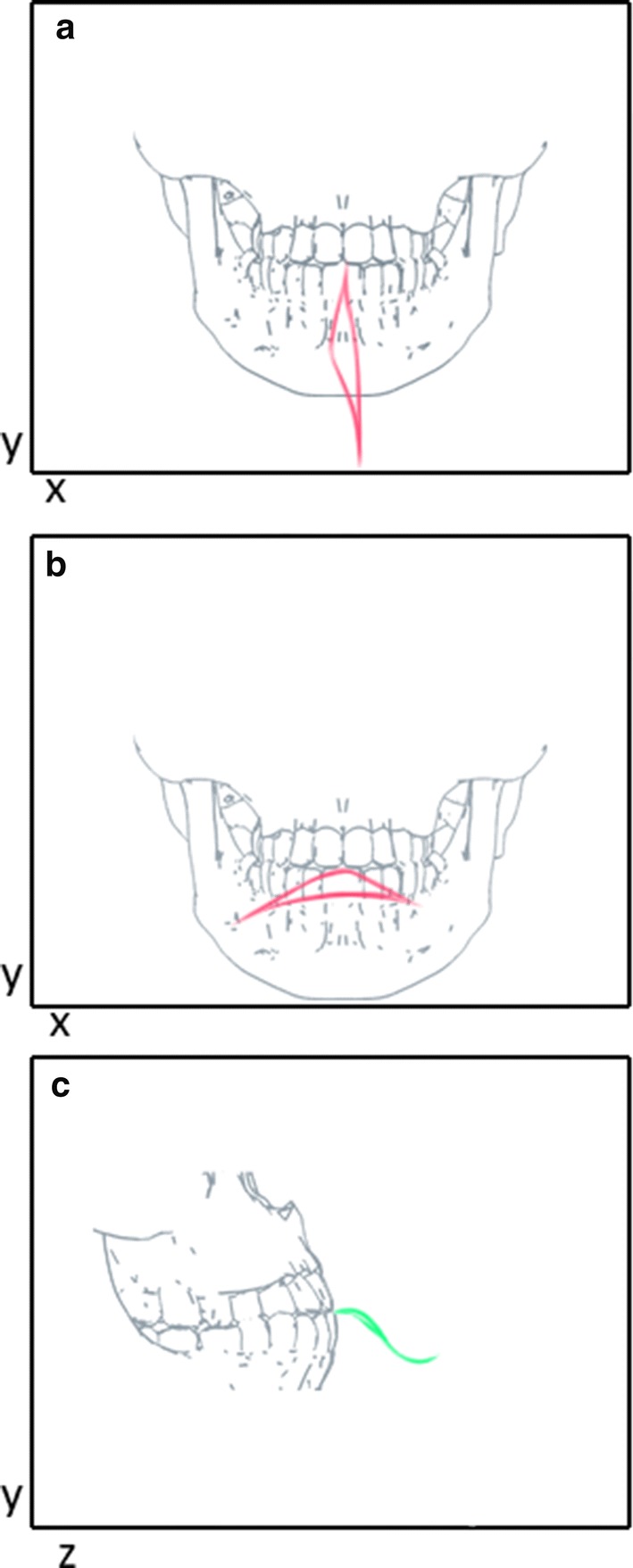
Movement trajectory for a person in the AG group (yellow dashed line), with the primary marker in blue: **a** opening–closing motion (frontal view); **b** lateral motion (frontal view); **c** protrusion/retraction motion (sagittal view). The red lines in **a**, **b** and the green lines in **c** show the trajectories of the six repetitions of each movement, showing their maximum points

Figure 1 shows the trajectory of the three motions studied (open/close of mouth; lateral movement of the mandible and protrusion movement) for an individual of the AG, using the jaw movement capture system designed in our laboratory.

**Fig. 2:**
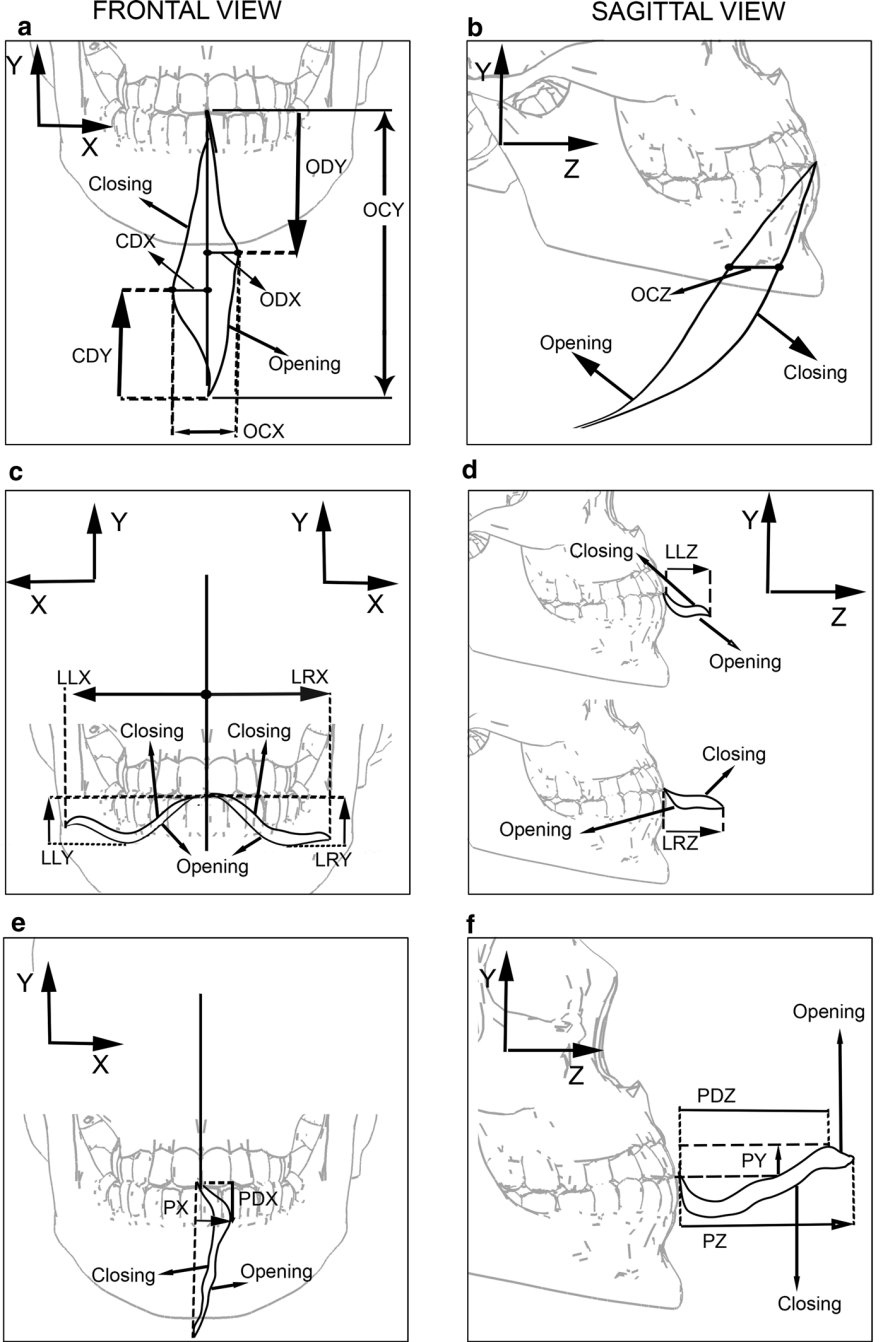
Measurements of the features extracted from each movement. The movements are indicated by arrows: **a**, **b** opening and closing movement; **c**, **d** lateral excursion to the left and to the right; **e**, **f** protrusion movement
- CDX—maximum deviation, in the x-axis direction, of the mouth closing movement (Fig. 2a);
- OCX—ODX + CDX (Fig. 2a);
- OCZ—maximum distance from mouth opening and closing trajectory in the z-axis direction (Fig. 2b);
- ODY—distance from the beginning of the mouth opening movement to the point where ODX occurs (Fig. 2a);
- CDY—distance from the beginning of the mouth closing movement to the point where CDX occurs (Fig. 2a);
- OCY—measure of maximum mouth opening in y-axis direction (Fig. 2a).

Through the data collected from the trajectory of the mandibular movement, the following features were calculated:

- ODX—maximum deviation, in the x-axis direction, from the mouth opening movement (Fig. 2a);

Features of the lateral displacement (right and left):

- LLX—maximum distance, in the *x*-axis direction, of the lateral movement of the mandible to the left side (Fig. 2c);
- LLY—maximum distance, in the *y*-axis direction, of the lateral movement of the mandible to the left side (Fig. 2c);
- LLZ—maximum distance, in the *z*-axis direction, of the lateral movement of the mandible to the left side (Fig. 2d);
- LRX, LRY and LRZ are analogous to LLX, LLY and LLZ, respectively, with jaw movement performed to the right side.

Features of the protrusion movement:

- PX—maximum distance, in the *x*-axis direction, of the protrusion movement of the mandible (Fig. 2e);
- PY—maximum distance, in the *y*-axis direction, of the protrusion movement of the mandible (Fig. 2f);
- PZ—maximum distance, in the *z*-axis direction, of the protrusion movement of the mandible (Fig. 2f);
- PDX—distance from the start of the protrusion movement to the point where PX occurs (Fig. 2e);
- PDZ—distance from the beginning of the protrusion movement to the point where PY occurs (Fig. 2f).

After calculating the number of individuals (*n*), it was found that the features from 10 individuals would be sufficient for the classification of all group pairs. The exceptions were the features OCZ, LLZ, LRY, LRZ, PX, PY, PZ and PDZ, which could not distinguish CG from AG; OCZ, which could not distinguish CG from MG; and ODX, LLX, LRX and PY, which could not distinguish AG from MG. Table 1 shows the features and situations in which the value of *n* (10) would not be able to distinguish CG from AG, CG from MG or AG from MG. Columns marked with “*” indicate features that require more than 10 volunteers to distinguish the group pairs analyzed.

**Table 1:** Features that require more than 10 volunteers to distinguish groups (marked with “*”)

Groups	Opening/closing features						
	OCX	OCY	OCZ	ODX	ODY	CDX	CDY
CG–AG			*				
CG–MG			*				
AG–MG				*			

	Laterality (left/right) features						
	LLX	LLY	LLZ	LRX	LRY	LRZ	
CG–AG			*		*	*	
CG–MG							
AG–MG	*			*			

	Protrusion features						
	PX	PY	PZ	PDX	PDZ		
CG–AG	*	*	*		*		
CG–MG							
AG–MG		*					

Although some features were unable to classify some group pairs with ten volunteers, these features were still retained. The decision to maintain these features was based on their importance for the classification of other group pairs. For example, the OCZ feature is not able to classify the CG–AG or CG–MG group pairs, but this feature is able to classify the AG–MG pair with the ten volunteers, as shown on Table 1. This same analysis was performed for all features that required more than 10 volunteers for the classification of group pairs.

Table 2 shows the evaluation results of the KNN, Random Forest, Naïve Bayes and Support Vector Machine methods in terms of sensitivity, specificity, precision and accuracy. The values shown in Table 2 were calculated using the following configurations:

**Table 2:** Evaluation of the classifiers for each group and comparison among KNN, Random Forest, Naïve Bayes and Support Vector Machine

Groups	Sensitivity (± STD)	Specificity (± STD)	Precision (± STD)	Accuracy (± STD)
KNN				
AG	0.9737 (0.0582)	0.9756 (0.0221)	0.9320 (0.0566)	0.9701 (0.0219)
MG	0.8703 (0.0793)	0.9897 (0.0142)	0.9676 (0.0425)	0.9599 (0.0221)
CG	0.9769 (0.0265)	0.9411 (0.0398)	0.9445 (0.0353)	0.9590 (0.0234)
Random forest				
AG	0.6852 (0.0030)	0.8839 (0.0011)	0.6642 (0.0021)	0.8342 (0.0009)
MG	0.6650 (0.0030)	0.8961 (0.0009)	0.6817 (0.0021)	0.8383 (0.0009)
CG	0.7900 (0.0018)	0.7951 (0.0018)	0.7947 (0.0014)	0.7926 (0.0011)
Naïve Bayes				
AG	0.7132 (0.0052)	0.8634 (0.0022)	0.6391 (0.0040)	0.8258 (0.0020)
MG	0.5601 (0.0056)	0.9424 (0.0018)	0.7716 (0.0055)	0.8468 (0.0018)
CG	0.8411 (0.0029)	0.7691 (0.0033)	0.7864 (0.0024)	0.8051 (0.0020)
Support vector machine				
AG	0.7942 (0.0049)	0.7894 (0.0045)	0.7925 (0.0040)	0.7918 (0.0037)
MG	0.7894 (0.0045)	0.7942 (0.0049)	0.7963 (0.0042)	0.7918 (0.0037)
CG	0.8846 (0.0030)	0.7718 (0.0050)	0.7989 (0.0037)	0.8282 (0.0029)

*STD* standard deviation

- KNN—K = 1;
- Random Forest—120 trees;
- Naïve Bayes—kernel (normal);
- Support Vector Machine—polynomial kernel.

The results in Table 2 show that the KNN classifier has a precision and accuracy of more than 93% and 95%, respectively, when differentiating between the three classes. The myopathic group had the lowest sensitivity value (87%). The Random Forest has a sensitivity and precision below 80%, but the CG classification showed similar findings across all evaluators (~ 79%). The Naïve Bayes classifier showed an accuracy of below 85%, while the MG identification sensitivity is the lowest found at 56%. The Support Vector Machine provides a similar classification with respect to the identification of AG and MG (~ 79%ç); however, it has a higher sensitivity (88%) and accuracy (82%) for the identification of CG.

**Fig. 3:**
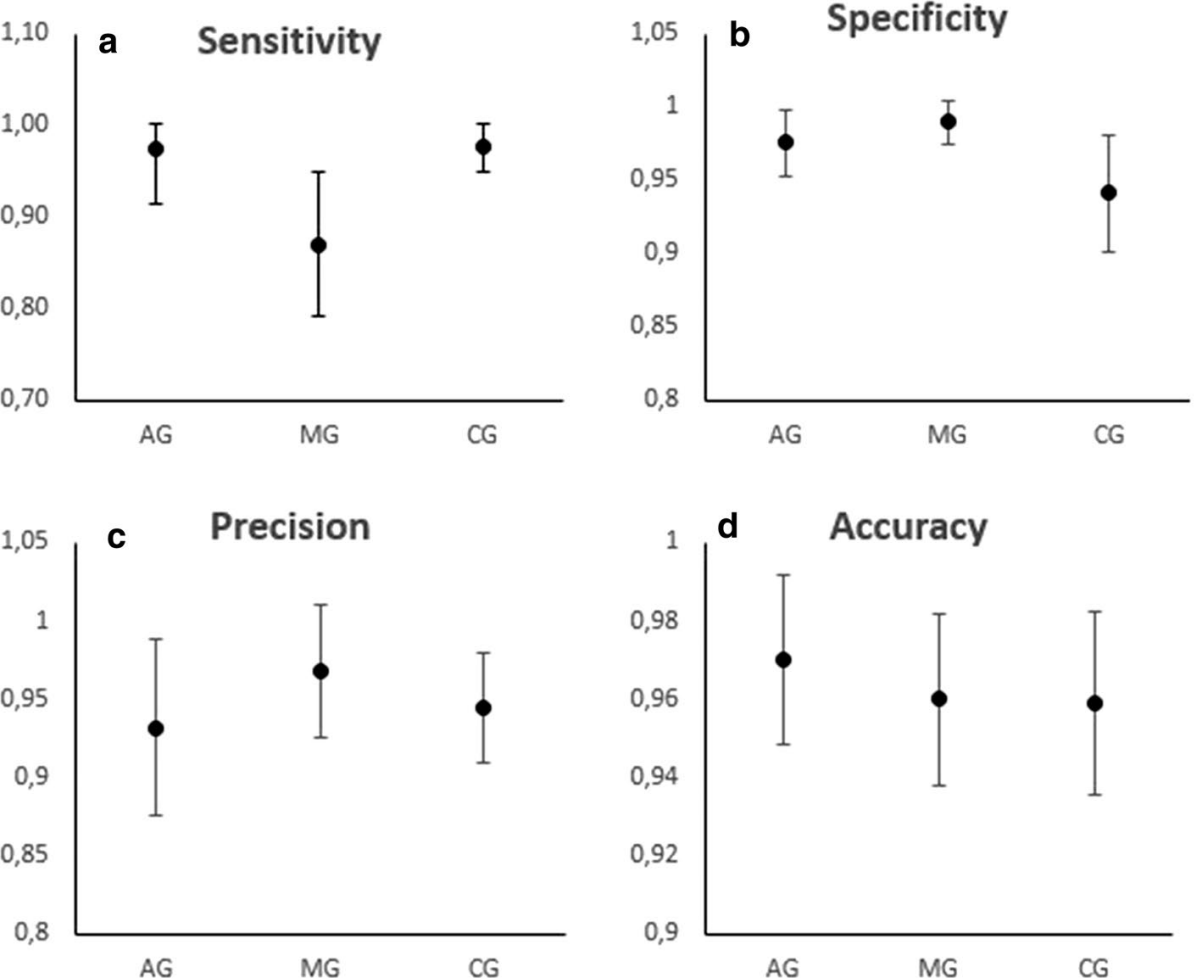
Evaluation of the KNN classifier: **a** sensitivity; **b** specificity; **c** precision; **d** accuracy

The results for sensitivity, specificity, precision and accuracy of the KNN classifier are summarized in Fig. 3.

## Discussion

### Challenges in the classification of TMD

Recent reporting of TMD has shown an increase in both severity and incidence [12]. TMD is the second most common musculoskeletal condition (after chronic low back pain), resulting in pain and disability [6]. TMDs affect the normal life of individuals and interfere with their daily activities. Correct diagnosis is crucial for receiving proper treatment and follow-up for that particular type of TMD. Qualitative approaches are typically used to assess and identify the TMD, which requires the training of a health professional in a clinical setting. In this sense, a quantitative method would help to improve and automatically evaluate an individual, leading to a more accurate diagnosis, independent of professional experience, particularly in the clinical context where such professionals are not readily available.

A wide variety of techniques can be applied to the diagnosing of TMD. The large number of diagnostic elements that examiners must take into account makes it essential to use an accurate TMD classification system [28]. Among the techniques used, AROM proved to be reliable for the diagnosis of TMD. However, this technique depends on the experience of the examiner and, unfortunately, the visualization of deviations during the movement of the mandibular along its trajectory is very difficult, especially in the early stages of TMD. However, the use of an optoelectronic system to record jaw movements achieves an accuracy and precision of 0.1 mm, in addition to recording the entire trajectory of the movement. Additionally, the AROM method, using conventional tools, is not able to classify volunteers with TMD from the AG and MG groups.

Although these methods can diagnose TMD with high sensitivity and high specificity, it is still a challenge to classify TMD as myopathy or arthropathy, as pain referred in TMD is more complex and more difficult to analyze and diagnose precisely due to the distance between the probable site of origin and the place where it is manifest [28]. In addition, the clinical presentation of TMD in patients is individually different. Thus, many patients do not fit into only one TMD category and other patients may not fit into any category [29].

The technique proposed in this paper allows for the automatic classification of individuals with TMD into groups AG and MG. This classification is only conceivable thanks to the possibility of using new features (due to the accuracy and precision of the optoelectronic system for the recording of jaw movements) other than the maximum opening of the mouth, maximum laterality and maximum protrusion.

### Evaluation of the features used

The method involves the analysis of four different movements of the jaw on three axes, a feature that enhances the precision of classification. From these movements and the analyzed plans, 18 features were chosen for analysis, in order to diagnose TMD and its subtypes (arthropathy or myopathy). Some of these features, as shown on Table 1, are not able to classify all group pairs with ten individuals. However, the same feature that cannot classify a group pair is capable of classifying another group pair. Thus, all the features contributed to the identification of the groups analyzed.

The features that presented the greatest difficulty in classifying the groups were those related to laterality (LLZ, LRY, LRZ, LLX and LRX). The authors Leeuw et al. also acknowledged this challenge in their study, reporting that there was no statistical difference between patients with TMD and the healthy group [30]. This finding also agrees with the study of Mazzetto et al. who used a 3D-ultrasonic system and a digital caliper rule to compare one group with TMD and another healthy group. They found no statistical difference between the groups in the lateral movements [31].

### Evaluation of the classifiers used

In this study, four classifiers were compared (KNN, RF, NB and SVM). The study by Haghnegahdar also used the same classifiers, but with two major differences. The first was that the study by Haghnegahdar used image data and the second is that the aim of work by Haghnegahdar was to separate healthy individuals from individuals with TMD, without classifying the individuals into the TMD subclasses (arthropathy and myopathy) [12].

The use of four types of data classification algorithms presented herein had the ability to demonstrate their performance results. The KNN classifier has an acceptable precision and accuracy rate (> 90%). The DC/TMD for clinical and research applications [6] states that the acceptable sensitivity and specificity for a definitive diagnosis is ≥ 70% for the former and ≥ 95% for the latter. Our method shows a > 97% for sensitivity and > 97% of specificity when classifying the individual as AG and > 87% and > 98% for individuals with MG.

The KNN classifier presents the best results when compared to RF, NB and SVM. This result may be due to the fact that KNN is a non-parametric tool, the adaptive nature to the dataset in the forming of nonlinear boundaries for the data points, along with demonstrating its capabilities as a tool for approximating a point to a dataset [32–34].

KNN is relatively simple to use and does not require professional experience in patient preparation. However, the drawback of the proposed method is that it requires a motion capture system that is not always readily available.

## Conclusions

We analyzed the human jaw motion trajectory for three different movements and developed a data analysis method as an auxiliary to the automatic diagnosis of TMD. Individuals with and without TMD were divided into three groups (CG, MG, AG) and the KNN classifier was the most capable with regard to separating each individual into clusters at an acceptable rate. The approach suggested herein has been able to discriminate between healthy individuals and individuals with TMD and identify these as individuals with myopathic diseases attributable to arthropathic disorders. The study has a statistical demonstration that is consistent with the literature and the proposed method can be used to assist the professional in the diagnosis, classification and follow-up of individuals with TMD.

## Methods

### Selection of individuals

**Fig. 4:**
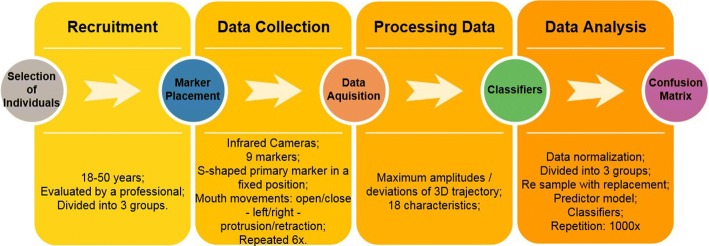
Methodology divided into four stages: recruitment, data collection, processing data and data analysis

Forty individuals were selected for this study, ranging from 18 to 50 years of age. All participants were informed of the tests and tasks of the study and signed a consent form. The Human Research Ethics Committee approved all protocols (Human Research Ethics Committee approval ID: 164/10 and 318.962). This study followed the steps shown in Fig. 4.

Initially, the participants are assessed by an experienced professional, a DC/TMD questionnaire specialist, and classified into three categories according to the results: control group (CG) (20 healthy individuals, 5 males and 15 females); myopathic disorder group (MG) (10 individuals, 3 males and 7 females); and arthropathic disorder group (AG) (10 people, 4 males, and 6 females). Highlighted here is that the DC/TMD questionnaire does not list TMD in the category of severity [35].

A pilot study was conducted with 12 individuals (4 CG individuals, 4 AG individuals and 4 MG individuals) to calculate the sample size. The same features used for group classification were extracted from these individuals. Each feature was re-sampled 1000 times using bootstrap, and the mean and standard deviation were determined from each feature. This procedure was carried out for the three groups (CG, AG and MG). From the means and the standard deviation, the number of individuals required to classify each group was calculated using Eq. 1 [36]:

1 $$ n = 2*\frac{{\left( {z_{\alpha } + z_{\beta } } \right)^{2} *\sigma^{2} }}{{(\mu_{1} - \mu_{2} )^{2} }}, $$

where, *n* is the number of individuals required; *z*_*α*_*(*obtained from the normal curve table), the *z*-score value for *α* = 0.05 in a two-tailed test (*z*_*α*_= 1.96); *z*_*β*_*(*obtained from the normal curve table), the *z*-score value for a power of 80% (*z*_*β*_= 0.84); *σ*, the standard deviation; and *µ*_1_ and *µ*_2_ are the mean of the features of the groups under evaluation.

### Data collection

A motion-tracking system consisting of infrared cameras (OptiTrack Flex V100; Natural Point, Corvallis, OR, USA) was used to capture the biomechanical features studied, which identified reflective markers located on the face of the individual. A set of nine reflective markers with a diameter of 10 mm were placed on the face of the individual.

**Fig. 5:**
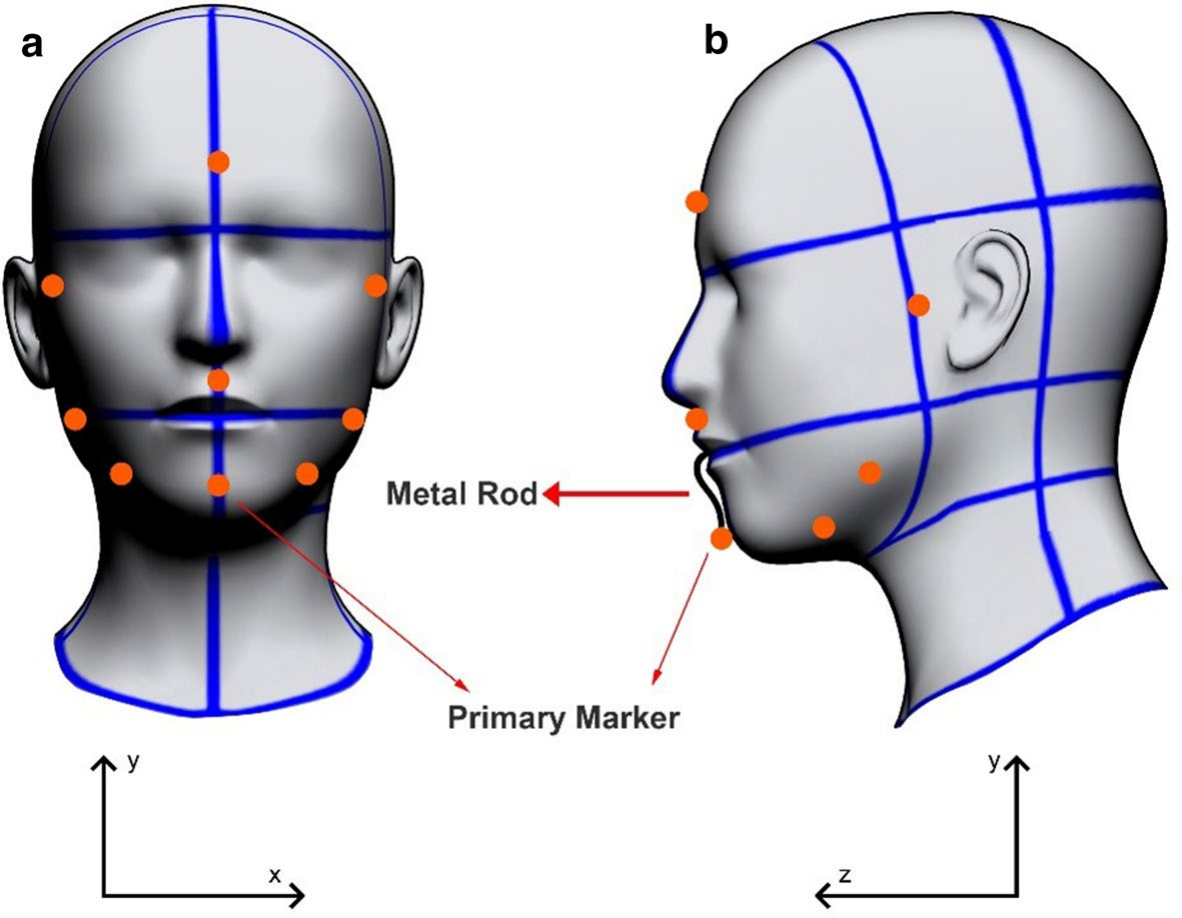
Marker positioning on the face of each individual: **a** frontal view, **b** sagittal view. The primary marker was placed on an S-shaped metal rod. The secondary markers were placed as follows: 1 on the forehead, 1 at each side of the temporomandibular joint, 2 at each side of the jawbone, and 1 in the middle of the labial philtrum

The jaw movement was tracked by means of a primary marker (a stainless steel wire with a tip made from a plastic sphere 10 mm in diameter covered by a reflective material) which was fixed to the lower incisors (Fig. 5a). The movement of the primary marker is therefore similar to the motion of the lower incisors and as such possesses the same movement as the previous region of the jaw. This marker has an S-shaped contouring around the mandibular incisors, thus, eliminating the compression of the upper and lower labial tissue, which could create interference in the movements of the jaw (Fig. 5b). The primary marker was fixed with a zinc oxide eugenol paste, not interfering with dental occlusion while in movement. The marker was sterilized after each use. The reflective marker placed on the head, functioned as a reference for decreasing the impacts in measurement errors, due to head movements. The other 7 reflective markers (10-mm-diameter plastic sphere fixed by Velcro tape) were used solely for the purpose of enhancing the visibility of mandible motions, without any influence on the data collection of movements.

Each individual was seated in an upright position on a normal chair with a headrest. The individual was positioned parallel to the ground in the Frankfurt horizontal plane. Three infrared cameras (OptiTrack Flex V100-Frame Rate 100 Hz) were positioned on tripods as follows: the front camera was positioned 1.3 m away and the other two cameras were positioned 1 m away, forming a 60° angle together with the front camera. The three cameras were placed 20 cm above the headline.

Before the data were recorded, each individual was given training. The set of tasks involved maximum range of motion for elevation/depression (opening/closing movement of the mouth), left and right lateral movement of the jaw (closing of the mouth) and protrusion/retraction (closing of the mouth). The tasks followed international parameters for the evaluation of the temporomandibular joint for physical examination.

In order to determine how many times the tasks should be repeated, a pilot study was conducted with 12 subjects. The results showed that with six repetitions, the average for the features changed very little. Thus, each task was performed six times. The speed of each task was performed at the discretion of each individual.

A custom software for jaw motion capture, designed in our laboratory [20], was used to determine the trajectories of the markers in the tasks described and these were exported as numerical data for further analysis. It is important to note that, despite the multiple images coming from all cameras, the image-tracking system software converts all collected images into one image, where the motion will be analyzed. The analysis uses a band-pass Butterworth filter with 4 poles and the cut-off frequencies are 0.01 Hz and 8 Hz, since mandible voluntary movements have a frequency of 6–7 Hz [20].

### Feature selection

There is currently no standard for the features of mandibular movement. However, the AROM technique recommends some mandibular movements that will be used and, from these movements, some features will be selected.

The movement performed by the patient is an important step for collecting the trajectory and for selecting the features. In this way, the movements requested from the volunteers for collecting the trajectory were put into four types: opening/closing of mouth (OC); lateral movement of the mandible to the left side (LL); lateral movement of the mandible to the right side (LR) and protrusion movement (P).

In this way, the 3D trajectory of the requested movements was collected using the infrared camera system. From the 3D trajectory, features were selected on the three axes (*X*, *Y* and *Z*). To prevent irrelevant movements from influencing the analyzed features, maximum displacement was used. In this way, the features chosen consisted of the distance (in millimeters) of the maximum displacements of the jaw movements in 3D-*XYZ* axes of each trial and the lateral deviation of the mandible in relation to the axis of each movement.

The maximum displacements of the four movements give rise to 12 features (four movements and three axes). In addition to these features, the maximum deviations that occurred in the trajectory were used, such as the maximum lateral deviation of the opening movement (OD) on the plane *XY*, maximum lateral deviation of the closing movement (CD) on the plane *XY* and the maximum lateral deviation of the protrusion movement of the mouth (PD) on the plane *XZ*. Thus, 18 features were used (Table 3).

**Table 3:** Features extracted from each movement

Movement	Features	Lateral deviation
Open/close	OCX/OCY/OCZ	ODX/ODY/CDX/CDY
Lateral left	LLX/LLY/LLZ	
Lateral right	LRX/LRY/LRZ	
Protrusion	PX/PY/PZ	PDX/PDZ

### Prediction and validation

Data were normalized using *z*-score. For normalization purposes, the z-score is used, in order that each point in the dataset has the same scale. The formula for *z*-score normalization of a given value is:

2 $$ zs = \left( {\frac{{\left( {{\text{value}} - \mu } \right)}}{\sigma }} \right), $$

where $\mu$ is the mean value of the feature and $\sigma$ is the standard deviation of the feature.

Following this, the data were separated into the three groups (AG, MG, CG). Prior to the application of a classifier, two subsamples were created for each of the three groups (AG, MG and CG), one subsample for creating the model and the other subsample for validating the model created for the performed classification. For the model subsamples, the original data were randomly re-sampled 1000 times with a replacement sample, each subsample comprised about half of the original data set. The same procedure was performed for creating the validation subsamples.

To create a predictor model for each group, four kinds of classifiers were employed using the model subsamples of each group: K-nearest neighbor classifier (KNN) [26, 37], using an Euclidean distance of 1, Random Forest classifier using bootstrap aggregated (bagged), the Naïve Bayes used a Gaussian distribution and the SVM uses a one-vs-one approach. These classifiers were chosen as they are the most widely used in machine learning with good results. Moreover, due to their wide use, they are already well known [12].

The KNN is well known and is used as a method with no prior assumptions about how data are distributed [38–40]. When a data point is given, KNN searches the training dataset for its K-nearest samples closest to the data point using Euclidian distance, given by Eq. 3, between a point $A\left( {a_{1} ,a_{2} , \ldots ,a_{n} } \right)$ and $B\left( {b_{1} ,b_{2} , \ldots ,b_{n} } \right)$. For an optimal response to this technique, tests were performed on the *K* value ranging from 1 to 4. The best results were achieved with *K* = 1, with this being the value used to classify the TMD.

3 $$ d\left( {A,B} \right) = \sqrt {\left( {\mathop \sum \limits_{i = 1}^{n} \left( {A_{i} - B_{i} } \right)^{2} } \right)} $$

Random Forest is an algorithm that uses a combination of individual tree predictors [23, 41]. To choose the best number of trees, the data were processed with the number of trees ranging from 60 to 1000. The best results were found with the number of trees equal to 120.

The support vector machine tries to classify data by the use of a separating hyperplane [42, 43]. SVM can use three types of kernels to classify the data, namely the linear, Gaussian or polynomial kernel. The data were classified using the three types of kernels. The best results were achieved with the polynomial kernel.

The Naïve Bayes classifier is based on the Bayes theorem, which assumes independence from the predictors. In order to perform classification, the Naïve Bayes classifier can be used with Gaussian, multinomial or kernel (normal) predictors. Tests were performed with predictors, and the kernel (normal) predictor showed the best results.

After the creation of the model, a predictor for each classifier was applied to determine the probability that an individual would be in class AG, MG or CG and validate the model.

### Statistical analysis

To evaluate the result of the classifiers, the scores calculated were sensitivity, specificity, precision and accuracy. Sensitivity is a measure of how many positives were correctly classified as positives (Eq. 4). Specificity is a measure of how many negatives were correctly classified (Eq. 5). Precision is the frequency of when a positive condition is correctly classified (Eq. 6). Accuracy is based on frequency, which generally states the classifier is correct (Eq. 7):

4 $$ {\text{Sensitivity}} = \frac{TP}{TP + FN}, $$

5 $$ {\text{Specificity}} = \frac{TN}{TN + FP}, $$

6 $$ {\text{Precision}} =  \frac{TP}{TP + FP}, $$

7 $$ {\text{Accuracy}} =  \frac{TP + TN}{TP + TN + FP + FN}, $$

where *TP* is the number of true positives, *FN* is the number of false negatives, *TN* is the number of true negatives, and *FP* is the number of false positives. On a multiclass confusion matrix, the values of *TP*, *FP*, *FN*, *TN* are given by:

8 $$ TP_{i} = c_{ii} , $$

9 $$ FP_{i} = \mathop \sum \limits_{l = 1}^{n} c_{li} - TP_{i} $$

10 $$ FN_{i} = \mathop \sum \limits_{l = 1}^{n} c_{il} - TP_{i} , $$

11 $$ TN_{i} = \mathop \sum \limits_{l = 1}^{n} \mathop \sum \limits_{k = 1}^{n} c_{lk} - TP_{i} - FP_{i} - FN_{i,} $$

where $c_{il}$ is the position of the element $i$ on the matrix.

After the application of the four methods, a subsequent matrix with the resulting scores of each validation subsample was created. Each score possessed 1000 values, calculated from each validation subsample. In terms of validating the results for the classifiers, the average and standard deviation were calculated from each score.

## Data Availability

The datasets generated in the current study are not publicly available due to the ethical restrictions preventing public sharing of data. A non-identified set may be requested after approval from the Review Board of the Institution. Requests for the data may be sent to the corresponding author.

## Abbreviations

TMD: Temporomandibular disorder
TMJ: Temporomandibular joint
DC: Diagnostic criteria
CT: Computed tomography
MRI: Magnetic resonance imaging
KNN: K-nearest neighbor
SVM: Support vector machine
NB: Naïve Bayes
RF: Random forest
CG: Control group
MG: Myopathic disorder group
AG: Arthropathic disorder group
OC: Free opening/closing of mouth
LL: Lateral movement to the left side
LR: Lateral movement to the right side
P: Protrusion movement (P)
OCD: Deviation of the opening
LD: Deviation of the closing
PD: Deviation of the protrusion of the mouth
TP: True positive
FN: False negative
TN: True negative
FP: False positive
STD: Standard variation
EMG: Electromyography
sEMG: Surface electromyography
